# Eco-Efficiency in the Polymeric Packaging Sector: Production Planning and Control Strategies for Economic and Environmental Gains

**DOI:** 10.3390/polym17091131

**Published:** 2025-04-22

**Authors:** Itamar de Souza Costa, Geraldo Cardoso de Oliveira Neto, Rodrigo Neri Bueno da Silva, Sergio Ricardo Lourenço, Leonardo Ribeiro Rodrigues, Marlene Amorim

**Affiliations:** 1 Industrial Engineering Post Graduation Program, Federal University of ABC, São Bernardo do Campo, São Paulo 09606-045, SP, Brazilrodrigo.neri@cummins.com (R.N.B.d.S.); sergio.lourenco@ufabc.edu.br (S.R.L.); ribeiro.rodrigues@ufabnc.edu.br (L.R.R.); 2 Department of Economics, Management, Industrial Engineering and Tourism (DEGEIT) and GOVCOPP, University of Aveiro, 3810-193 Aveiro, Portugal; mamorim@ua.pt

**Keywords:** polymer scrap, production planning and control, eco-efficiency

## Abstract

Production Planning and Control is directly involved with production activities in the polymer packaging industry, aiming to produce more with fewer resources. This reduces polymer scrap, generating positive results in economic and environmental terms. The importance of reducing polymer waste during the extrusion process and the use of theoretical models and simulation tools, along with recycling and reprocessing practices, emerges as an effective strategy to improve production and minimize environmental impacts. This study aims to evaluate the economic and environmental gains of adopting activities/tools used in production planning and control in the polymer packaging industry. The research method adopted was a case study conducted through semi-structured interviews. It was concluded that polymer scrap in the production system was reduced through actions taken by production planning and control, contributing to the micro-level circular economy, generating significant economic and environmental gains.

## 1. Introduction

Production Planning and Control (PPC) is the sector responsible for performing strategies using tools/activities in order to manage, organize, control, and monitor processes in the transformation of inputs into finished products [1]. In this way, the PPC has always aimed at economic gains through efficient production. However, unrestrained production to meet increasing demand from consumers has contributed to the degradation of ecosystems [2].

Population growth, urbanization, and industrialization are related to the high level of consumption of industrialized products, which lead to a growing demand for natural resources and cause serious consequences for the environment and for the human being [3]. As a result, any industrialized product has a negative impact on the ecosystem, whether due to the production process, raw material, or incorrect disposal of the product after its use [4].

The growing concern regarding the environmental impacts of the plastics industry has driven the search for solutions that minimize material waste and improve the sustainability of production processes. In the packaging industry, low-density polyethylene (LDPE) and linear low-density polyethylene (LLDPE) are widely used, particularly in processes such as extrusion, for the production of plastic films, tubes, and other products. However, although these materials are extensively used due to their versatility and low cost, they generate large volumes of waste during production, which poses a challenge both for economic efficiency and for the environment [5,6].

The industrial benchmarking of companies in this sector indicates that, on average, waste in plastic film extrusion processes ranges from 2% to 9% of total production when using LDPE and LLDPE. Thus, considering a daily production of 10 tons, the waste generated by LDPE would be around 0.5 tons, and for LLDPE, approximately 0.8 tons. In this scenario, with four daily stoppages for setup/waste removal, each stoppage would represent around 200 kg if LLDPE is the processed material. The average values reported are supported by [7], who states that average losses per stoppage in LLDPE extrusion setup operations are around 6.7%.

Polymer extrusion involves transforming the material into different shapes through heating and the application of pressure. During this process, variables such as temperature, pressure, and speed must be rigorously controlled, as deviations from these parameters can result in defects in the final product and an increase in waste. In this context, the adoption of Production Planning and Control (PPC) strategies stands out as an effective approach to optimize these processes, reduce polymer waste, and enhance operational efficiency. PPC aims to adjust production variables in real time, enabling the minimization of waste and improvement of economic outcomes.

This study, the adoption of PPC practices/tools promoted eco-efficiency, an aspect little explored in the scientific literature. Thus, PPC is the main method in the context of production management. Although other methods may be considered alternative or complementary, depending on the analytical perspective, they are not the focus of this research. Alternative examples include Lean Manufacturing practices/tools, such as Kanban, 5S, and Kaizen; Just in Time (JIT); Optimized Production Technology (OPT); and the Theory of Constraints (TOC), which is frequently applied in PPC activities in organizational practice. PPC is the central part of the management and optimization of production systems. Thus, employing eco-efficiency practices/tools directly in PPC will yield satisfactory results. Some advantages can be listed: (i) eco-efficiency practices/tools are listed in strategic production planning; (ii) eco-efficiency practices/tools are established directly in the production schedule in interface with the shop floor activities; (iii) eco-efficiency practices/tools are considered in the control of the production system, allowing the listing of performance indicators with visual management for the reduction of environmental impacts.

In addition to its economic benefits, the implementation of PPC practices in the packaging industry can also contribute to environmental sustainability. By promoting the more efficient use of materials, PPC supports the reduction of the environmental impact associated with polymer production, aligning with the principles of the circular economy. The integration of practices such as recycling and material reuse in the production process not only enhances resource utilization but also reduces the amount of waste generated, promoting cleaner and more sustainable production.

This study aims to evaluate the impacts of adopting PPC strategies on reducing polymer waste, such as LDPE and LLDPE, and their effects both in economic and environmental terms. Through a detailed analysis, the study seeks to understand how PPC practices can contribute to improving efficiency in the packaging industry while simultaneously minimizing the environmental impacts associated with this sector.

For Michelsen [8], the concept of eco-efficiency is a combination of economic and environmental efficiency that occurs in organizations. While for the World Business Council for Sustainable Development [9], eco-efficiency is achieved through products and services that satisfy human needs at competitive prices, reducing ecological impact and resource intensity throughout its life cycle.

Betts [10] mentioned that environmental decision-making strategies in organizations provide opportunities for generating competitive advantages. Therefore, companies that manage to keep their activities geared towards eco-efficiency will have a competitive advantage compared to their competitors. This search for minimizing environmental impact shows that CFP managers need to adopt tools and activities that provide the reduction of waste and the use of natural resources [11].

Oliveira Neto [12] contributed to the development of manufacturing paradigms, which he called incremental change through cleaner production and eco-efficiency. Companies that had only economic gains as their goal needed to adapt to the new scenario to reduce environmental impacts. Therefore, the PPC needs to adapt to these changes and start considering solutions in its planning to improve industrial eco-efficiency.

However, Ravi [3] mentions that the need to improve eco-efficiency in organizations becomes a challenge to identify alternative solutions, both for economic and environmental performance, without a trade-off between both. Oliveira Neto and Lucato [4] affirm that convincing entrepreneurs to practice production with environmental education has been challenging, since most of them aim to obtain only economic advantages, not being aware of the need to make these economic goals compatible with environmental protection. Thus, the PPC is the sector responsible for organizational activities that can increase industrial eco-efficiency in companies through the promotion of activities that reduce environmental impact with the use of fewer natural resources and less waste generation. According to Jabbour [13], the eco-efficient PPC adoption can occur as waste is reduced through recycling, reuse, or remanufacturing, generating economic and environmental gains for the company.

With this in mind, [4] presented a procedure for adopting PPC with environmental education applied to machine load sequencing techniques using the workload control. This allowed demonstrating that the company, through the interference of the PPC professional, was able to obtain economic and environmental gains.

In addition, factors such as awareness of customers, consumers, and government requirements are also reasons that may lead the PPC to adopt activities/tools in its planning in order to minimize the consumption of electricity and water, among other natural resources [14]. Thus, the PPC can contribute through procedures for remanufacturing, recycling, reusing, and using renewable raw materials, providing a favorable path for industries, by adopting productive procedures aimed at achieving industrial eco-efficiency.

According to Brennan [15], there are forces at work, such as greater awareness of the environment by both the consumer and the producer, recycling regulations, and resource conservation needs. These changes lead to new challenges and a fundamental reassessment of the traditional manufacturing paradigm. Companies that are more focused on the future are acknowledging opportunities arising from this changing environment. In view of the current scenario, where inputs such as water and electricity, among other resources extracted from nature, are increasingly scarce, it is necessary to search for activities and tools that not only enable economic gains but also minimize the environmental impact.

According to Cannata [16], the PPC must include energy-efficiency performance indicators in its activities, since electric energy corresponds to 33% of global energy consumption and 38% of total CO_2_ emissions in manufacturing companies. PPC professionals need to start rethinking and interact with environmental practices in a positive way in order to increase economic gains, reducing environmental impacts, saving energy, recycling, facilitating remanufacturing, as well as replacing environmentally harmful raw materials with other biodegradable or renewable sources.

Although there are several studies in the literature that address principles to make industrial processes increasingly sustainable, it is clear that there is a lack of environmental practices and guidelines capable of providing details according to each industrial process [14,17]. As a result, there are few studies that link PPC and eco-efficiency. In the literature search carried out as part of this work, only five studies that fit these criteria were found, and in all of them the methodology used was the case study.

Plehn [18] carried out a case study in Switzerland that had the implementation of production sequencing as a PPC practice. The measure allowed to reduce the setup time and consumption of electricity by 13%. Goggin [19] carried out a case study developing improvement in the information management of the end-of-life product. Information sharing along the chain improved the demand forecast and the master production plan, making remanufacturing more predictable. Cao [20] also developed ways to maintain product life cycle information on an RFID tag, allowing greater support to the PPC regarding uncertainties in the remanufacturing process. Wu [21], in turn, carried out a case study in Taiwan that, through multicriteria analysis, aimed at maximizing production capacity and reducing the cost of waste and water, in addition to minimizing the cost of inventory.

Finally, it was the only one that proposed a procedure to be used by the PPC through which it was possible to obtain simultaneously a significant reduction in environmental impact and also in cost. However, this work showed the application of the proposed PPC concepts through a case study developed in the chemical industry [4].

As the literature review showed, there are no previous studies that have explored the use of PPC as a tool to reduce environmental impacts and simultaneously improve costs in the polymeric packaging industry—the gap in the paper developed here. Working with this scenario, this work proposed to use the same procedures to perform economic and environmental analysis through practices of PPC [4] in polymeric packaging manufacturers, posing, as a result, the following research question: How can the activities and tools used by PPC generate eco-efficiency improvements in polymeric packaging manufacturing companies?

To answer this research question, a case study was carried out that allowed the identification of procedures and tools used by the PPC to obtain and measure the reduction of environmental impacts while improving costs, that is, improving the level of eco-efficiency of the studied company.

Through this study, we sought to generate contributions to the theory, as it shows the use of PPC to improve the eco-efficiency of companies in the polymer sector, thus generating knowledge hitherto not existing in the literature. From a practical point of view, this work shows company managers, especially those responsible for the PPC, how an adaptation of traditional practices in this area can generate improvements in the level of eco-efficiency for their respective companies.

The remaining part of this work is structured as follows: the next item presents the theoretical foundation that supports this study. This is followed by the presentation of the methods used and the results obtained in the case studies carried out. These results are then discussed, ending with the conclusions, contributions, limitations, and suggestions for future research.

## 2. Theoretical Foundation

### 2.1. Reduction in the Volume of Polymer Scrap

The growing concern regarding the environmental impacts of polymer waste has driven research and the implementation of strategies to minimize waste during manufacturing processes, particularly in extrusion. Polymer waste can occur at various stages of production, ranging from failures in controlling variables during extrusion to the disposal of non-conforming material or by-products. Reducing the volume of waste is, therefore, a priority for industries aiming to improve production efficiency, reduce costs, and simultaneously minimize environmental impacts.

The polymer extrusion process, such as for low-density polyethylene (LDPE) and linear low-density polyethylene (LLDPE), is particularly susceptible to variations that can generate waste. Several factors, such as temperature, pressure, extrusion speed, feed rate, and cooling, directly influence the quality of the final product and the amount of waste generated [5,6].

Polymers with inadequate flow properties can result in product formation failures or inconsistent extrusion, leading to excess material or defects [6]. Thus, precise temperature control is crucial. The processing temperature must be carefully regulated; however, if thermal control is ineffective, extrusion may result in compliance defects or poorly formed areas, generating waste [5].

Furthermore, the pressure applied during the extrusion process must be adjusted according to the polymer’s viscosity, the type of product to be extruded, and the characteristics of the extruder used. Inadequate pressure can lead to extrusion failures or material wastage. Another important factor is the residence time of the polymer within the extruder barrel, which affects both product quality and the amount of unused or wasted material [6].

The integration of theoretical models and simulation tools, such as LUDOVIC^®^ and Polyflow, has proven to be an effective approach to improving extrusion process efficiency and reducing waste. These tools allow extrusion parameters to be modeled and virtually optimized, simulating different production scenarios and adjusting variables without the need for physical experimentation. This not only contributes to the reduction of material waste but also optimizes energy consumption and improves the final product quality [5].

Moreover, the recycling and reprocessing of plastic waste have been identified as promising strategies to reduce waste in the extrusion process. The reuse of recycled materials, such as extrusion by-products or post-consumer materials, can be an effective way to reduce the volume of waste generated. This is particularly relevant in a scenario where the amount of polymer waste is growing significantly, resulting in increasing pressure to implement sustainable solutions for managing these wastes [22,23].

Effective cooling control also plays a crucial role in waste reduction. After extrusion, the still-hot material needs to be rapidly cooled to preserve the shape and properties of the final product. If cooling is inefficient, product deformation may occur, leading to material disposal. Therefore, controlling and optimizing the cooling process can significantly reduce polymer waste while improving productivity [5].

Considering the factors identified as sources of losses, PPC, which oversees production parameters, enables the analysis of the process flow to identify which losses occur and how they impact the production flow. For example, optimization of the manufacturing process can be achieved by ensuring proper cooling of the material (preserving the shape and properties of the final product). These manufacturing variables also affect production variables, such as reduced task time and lower scrap rates.

In the context of PPC, such manufacturing gains are translated into production gains, as they lead to shorter cycle times, fewer events, and reduced system fluctuation amplitudes, which in turn improve productivity indicators. Moreover, once the system/process bottleneck is identified, any action taken to optimize this specific process/activity will have a positive impact on the entire production system that relies on this resource.

Finally, the implementation of chemical recycling techniques, such as pyrolysis and dissolution/reprecipitation, has shown great potential for treating plastic waste, such as LDPE and LLDPE, converting them into new materials or fuels, thus reducing the volume of waste generated in industries [24]. The adoption of these techniques, combined with a well-structured Production Planning and Control (PPC) system, can significantly contribute to waste reduction, promoting more efficient and sustainable production.

### 2.2. Actions of Production Planning and Control (PPC) to Improve Eco-Efficiency in Industry

A systematic literature analysis was carried out to identify what the current literature presents relating the PPC and eco-efficiency. For this, eight sets of keywords and their cognates were formulated based on the PPC theme and its relationship with eco-efficiency in industries. The goal was to identify economic and environmental gains due to the adoption of PPC practices/tools. The following words were used: (i) “production planning and control” “eco-efficiency”; (ii) “production planning and control” “environmental” “economic”; (iii) “production planning and control” “environmental” “financial” (iv) “production planning and control” “sustainable”; (v) “production planning and control” “environmental” (vi) “production planning and control” “economic”; (vii) “production planning and control” “financial”; (viii) “production planning and control” “sustainability”. These words were grouped in order to associate the CFP practices and tools with the reduction of the environmental impact and economic gain. These sets of keywords were applied to the following databases: Science Direct, Emerald, Wiley Library, Scopus, Proquest, Compendex, Scielo, and Taylor & Francis.

Initially, 1326 articles were identified, which, when extracted and analysed, showed that 203 were repeated and 411 contained the term “environmental”, but represented other compound words that were not related to the environment. There were also 87 articles in which only the topic PPC was listed without any relation to the environment and 75 in which the environment was mentioned but not in relation to the PPC. Finally, 45 articles contained the word “PPC” and 48 the word “environmental” only in the references. There were 396 articles left that represented several areas that were not related to industries and were not aligned with this research scope; 35 were books related to the researched subject. Therefore, of the 1326 articles initially selected, only 26 made a direct connection between PPC and eco-efficiency, and in most of these, only thematic research evidence was found.

For the purpose of better description and analysis, these 26 publications were subdivided into two groups, the first being related to recycling, reuse, and remanufacturing, and the second focused on the efficiency of the use of energy and water. The articles also showed evidence of a relationship between conventional PPC practices and environmental practices. As a result, it was possible to identify that conventional PPC activities can reduce the negative impact on the environment and obtain economic gains by minimizing waste and contributing to eco-efficiency, as shown in Table 1.

The first group with 16 articles emphasizes the adoption of recycling through remanufacturing actions: (i) recycling/reuse of waste and emissions; (ii) recycling/reuse of waste and emissions and reduction and non-generation of emissions and resources; (iii) recycling/reuse of waste and emissions and efficiency in the use of raw materials. Still in the first group, there is an article that can be divided among the groups, as it mentions: (iv) recycling/reuse of waste and emissions—the other part of the article deals with (i) energy use efficiency; (ii) efficiency in the use of energy and efficiency in the use of raw materials; (iii) energy use efficiency and reduction and non-generation of emissions and resources; (iv) energy use efficiency and water use efficiency; (v) energy use efficiency; (vi) efficiency in the use of water and reduction and non-generation of emissions and resources, making it possible to demonstrate the relevant results on environmental and economic gains in remanufacturing and manufacturing processes.

Initially, the practice of efficiency aimed at recycling/reuse and remanufacturing waste and emissions were addressed by 14 papers. Guide Jr. [26], found uncertainties in the remanufacturing that make PPC activities become complex compared to traditional manufacturing activities. In this way, a product designed for reuse was suggested in order to reduce lead time in the process and inventory costs due to waste during disassembly. It was also suggested an adaptation in the MRP that allows the management and control of the inventory in recoverable environments, in addition to providing waste prevention in a PPC conscious planning. Guide Jr. [27] carried out a survey and found characteristics in remanufacturing that require changes in the PPC activities. As a result, 60% of the surveyed companies found that lead time needs to be reduced, while 38% cited the lack of adequate systems. Most companies use hybrid systems like MRP combined with Just-in-time, Kanban, and DBR techniques. In response to economic questions, 78% of companies achieved sales in excess of $21 million per year, demonstrating that the remanufacturing process is economically and environmentally viable. In this context, remanufacturing closes the cycle with the use of materials forming an essentially closed manufacturing system.

Ferguson [29] developed an information system for decision-making in recycling end-of-life products, which allowed communication with access to data stored in various locations along the chain. And with that, it was possible for the PPC to carry out demand forecast, master production plan, and better manage the stock, with the possibility of analyzing costs for the purchase of end-of-life vehicles, in addition to reducing the environmental impact. Linton [30] developed a conceptual model with nine forms of product life extension that provides a guide to the differences and similarities, costs, and resources that are required in the adoption of the CFP by new ways of extending the product’s life. However, the inventory’s criticality, which is one of the problems faced by the PPC to balance demand with returns on remanufacturing, is reduced when considering the life span of the product, allowing environmental and economic gains. Tang [31] elaborated a mathematical model to define the execution time in the engine-disassembly process in remanufacturing and to determine a component-purchase strategy. The model allows better support for PPC to reduce inventory costs and reduce lead time, providing economic and environmental gains in the remanufacturing process.

Guide Jr. [25] developed capacity planning techniques for a remanufacturing system that has a high variation in its processing as well as in the recovery rate. This technique allowed for better resourcefulness compared to MRP capacity planning. Through remanufacturing, the author mentions that it is possible to estimate savings between 40% and 60% of manufacturing costs on a new product. Goggin [19] developed a resource recovery model through end-of-life product information along the chain. In this way, the PPC is able to develop improvements in its inventory management, demand forecast, and PMP, through information shared throughout its chain. Demand is planned annually, carrying out a master production plan on a quarterly basis and reviewed monthly, facilitating disassembly and making remanufacturing more predictable. This allows the environmentally correct disposal of waste, with attention to the prescribed treatment of toxic and hazardous waste

For Cao [20], uncertainties can be reduced by increasing the exploitation of information generated throughout the product’s life cycle. Through the developed software, product information and maintenance records can be evaluated to predict the status of quality, quantity, and turnaround time of used products. Thus, information on the product’s life cycle, as well as manufacturing, design, among others, is transferred to RFID tags, which allows better decision support to the PPC sector regarding the uncertainties in the remanufacturing process.

Digalwar [33] conducted a survey with 12 performance measures used in Green manufacturing. Among the performance measures found in the survey, the PPC is a sector that allows decision-makers to reduce costs in production processes and adopt strategies in response to current market needs. The research provides evidence related to the reduction of pollution through remanufacturing, recycling, and reuse, reducing material resources, thus minimizing the environmental impact.

Munot [34] cited three items in their theoretical work to emphasize the importance of remanufacturing: environmental regulations, awareness of customers about the green environment, and economic benefits. They also suggested projects for disassembly, in order to improve productivity and reduce lead time in the remanufacturing process.

Lage Jr. [36] proposed in their work a mathematical model to determine the best quantity to be disassembled in order to minimize the total cost in a remanufacturing system with stochastic routes. The shorter the higher cost route, the smaller the difference between costs, which justifies that, in some situations, disassembling more products than necessary to meet demand can result in a lower expected cost. The results contributed to a better PPC management practice using mathematical modelling in the master production plan to reduce environmental impact and also reduce costs in remanufacturing.

Guide Jr. [27], mentioned in their theoretical research that conventional supply chain management activities differ from managing remanufacturing supply chain activities in seven aspects, which increase uncertainties in PPC activities and in the remanufacturing process. In this context, MRP techniques can provide better control in the dependent demand stock, and by the same token, it has also been suggested to use information systems with new PPC techniques to reduce uncertainty in remanufacturing and keep them more predictable. As a result, the organization achieved greater savings and reduced environmental impact.

Jena [37] found that lately, researchers have been developing price policies to attract used products in order to reduce uncertainties in the remanufacturing process. With this, it was possible to maintain a balance between supply and demand, regarding the time and quantity in the return of used materials. Mawandiya [38] had as main objective to determine a stock policy to reduce costs in the acquisition of products in a closed-circuit supply chain that is part of a remanufacturing system. It was also possible to compare a traditional supply chain with a closed-circuit supply chain in remanufacturing, allowing us to conclude that the costs in the remanufacturing supply chain tend to be lower.

Nikolaidis [40] carried out work with eco-efficiency practices aimed at recycling/reuse waste and emissions and reducing and not generating emissions and resources. With the use of mathematical modelling, it was possible for the PPC to evaluate an economic lot of product purchases. An environmental project was also suggested with a rationalization of natural resources, reuse, reduction of waste, and, consequently, pollution reduction.

Only one survey was found through recycling/reuse practices for waste and emissions and efficiency in the use of raw materials. Prajogo [35] conducted a survey to study the extent and balance of the environmental management system diffusion within organizations in these areas: production, purchasing, sales, logistics, and R&D. One of the performances considered in the Green process was the PPC focused on reducing and optimizing waste. The research shows then the relationship between the PPC and eco-efficiency as evidence, demonstrating that the PPC is characterized by integration with practically all areas of the organization. This enables sharing and balanced diffusion of the Environmental Management System in different areas of the organization.

Therefore, with the use of some conventional PPC tools/activities, it was possible to verify qualitative evidence to promote improvement in recycling, reuse, and remanufacturing, such as forecast planning on the return of material for remanufacturing, using MRP to develop the master production plan, contributing to the system predictability improvement, and adopting mathematical modelling in the master production plan to reduce costs and environmental impact; planning to reduce the delivery time of remanufactured products through the development of the project for disassembly, repair, waste separation, assembly, final testing; taking into the account the life cycle of products when planning inventories to return of material actions after the end of their useful life, aiming at remanufacturing; adopting a mathematical modelling for the management of remanufactured products inventories; production capacity planning considering recycling and reuse, allowing the reduction of raw material costs; production performance control, allowing to minimize the waste of non-renewable materials; planning of production actions to optimize the system, reduce waste, and contribute to Green Manufacturing.

The second group presents 10 articles that relate the activities/tools of PPC with environmental and economic practices aimed at the use of energy and/or water efficiency. One study that mentions the efficiency of energy use and recycling/reuse of waste and emissions was identified. Brennan [15] developed a conceptual model and found that remanufacturing in terms of disassembly and assembly represents a challenge for the PPC. Design products for disassembly and recycling is suggested then, in order to minimize the consumption of electricity. As a result, due to the reuse of parts and cuts in the consumption of electricity, it is also possible to reduce lead time and minimize pollution in the environment.

Three studies regarding energy efficiency and efficiency in the use of raw materials were found. Grauer [39] developed decision support software that has an interface with the ERP to be used in multiple decision problems faced by the CFP manager. It allows the increase of produced thermoplastics quantity and quality, besides reducing the consumption of electric energy and providing solvent recycling in the production process, which, in turn, generates cost reduction and minimizes the environmental impact. Vollmer [41] implemented software integrated with ERP that allowed the calculation of material resources and energy-consumption data, making it possible to form indicators for the control of the PPC through the analysis of the life cycle and, as a result, improving operational, economic, and environmental performance. Angulo [42] complemented the integration of the ERP system with information and communication technology through the Factory Eco-Mation architecture. It integrates data from environmental sensors, production information, and energy-consumption records, allowing the PPC, aiming at improving the Environmental Management System, to manage and make decisions based on economic and environmental performance.

Two studies were identified on energy efficiency and reduction and non-generation of emissions and resources. Cannata [16] found in their literature review that the PPC’s challenge is to face the adequate inclusion of energy efficiency in production since 33% of global energy consumption and 38% of total CO_2_ emissions are due to manufacturing. With this, actions such as sequencing rules, avoiding production during peak energy hours, and performance evaluation, among other activities of the PPC, can help reduce energy consumption and the environmental impact.

In the work of Plehn [18], the production sequencing was implemented to reduce the setup time and minimize the consumption of CO_2_ and energy by 13%. It is thus important to sequence the loads correctly to reduce energy and CO_2_ consumption, as it depends not only on the production volume, but also on the type of machine used and the proper grouping of the processed materials types.

Two studies were identified in the relationship between efficiency in the use of energy and water. Nikolopoulou [40] found that it is possible to create stochastic methods for decision-making to analyze the environmental impact in chemical processes. However, this fact is limited not only to the material involved in the process, but also to factors such as the analysis of energy and water consumption and analysis of the product life cycle, which are complex analyses to be carried out in remanufacturing and recycling processes in the supply chain. This complexity is due to the uncertainty in quantity, lead time, and inventory generated by the remanufacturing process. Only one survey related to the efficiency in the use of water and reduction and non-generation of emissions and resources. Oliveira Neto [4] developed a case study in the chemical industry located in Brazil and found that the application of load sequencing through workload control resulted in a 42% cost reduction. The research also concluded, through the application of the Material Intensity Factors (MIF) tool, that there was a significant reduction in the environmental impact.

Only one study was identified regarding eco-efficiency practices aimed at reducing and not generating emissions and resources and efficiency in the use of water. Wu [21] carried out a case study in a textile dyeing company in Taiwan and concluded that the use of multicriteria analysis can assist the PPC manager in making environmental and economic decisions, allowing them to minimize the production and environmental costs. It also aims to maximize production capacity and reduce the inventory and the cost of waste and water.

Only one study of energy efficiency practices was found. Mousavi [43] were able to demonstrate that it is possible for the PPC to improve energy efficiency when dealing with dynamic units in the production process, in order to obtain results of improvement in eco-efficiency. In this way, the total production time and yield are related to economic aspects, while energy consumption is characterized by an environmental impact.

Therefore, the PPC was able to promote improvements in eco-efficiency using its tools/activities by adopting the sequencing of production to reduce electricity and minimize water consumption in the production process. It was also possible to integrate the ERP/MRPII system with Information and Communication Technology to measure eco-efficiency indicators. In addition to considering, in PPC planning, the multi-criteria decision model applied to capacity planning, including inventory cost, production cost, environmental costs, and energy efficiency.

## 3. Methods

This section presents the research methodology that was used to carry out this work, in which a systematic review was developed to identify theoretical contributions and research gaps, followed by a field study with an exploratory objective through the case study, using documentary research and semi-structured interviews as a data-collection technique.

### 3.1. Case Study Method

The case study method was chosen because this research attempts to answer questions related to “how” and “why”. Likewise, it is also exploring a contemporary phenomenon in a practical context where the boundaries between the phenomenon and the context are unclear [44]. To select a company to support the case study, Patton [45] suggests the use of intentional sampling, this being the case in which the researcher can extract a substantial amount of relevant information on the central issues under evaluation. Among the several strategies recommended by Patton [45] for choosing intentional cases, this work uses the sampling of typical cases. In these, the company to be selected for the case study must present a situation in which it is possible to identify actions taken by the PPC area that has resulted in an improvement in the level of eco-efficiency of the studied company, that is, registered gains both in the economic and environmental spheres.

### 3.2. Data Collection

For data collection in the case studied, the semi-structured interview technique was chosen, as it is considered the most suitable for data collection in qualitative research [46,47]. To ensure that all required information was obtained during the interviews, an aide-memoire was prepared with the main facts to be verified. The main topics covered in the interviews included: (a) general history of the company (general information about the company, its profile, ownership, and its brief history); (b) contextual information about PPC and plant operation before implementing the procedure suggested by this work, including the data necessary to calculate the level of eco-efficiency and the environmental impact of resources; and (c) the same set of information after implementing the changes provided for in the procedure analyzed here. That represents then a “before and after” case study [48].

Thus, the research procedure of the present work comprises the following steps: (i) Establish the research objectives; (ii) Identify the industries that fit the theme and objectives; (iii) schedule an interview with the legal representative, who will be able to provide data and information for carrying out the research; (iv) through documents, interviews, and observations, understand the flowchart of the production process and describe the PPC actions adopted that have generated economic and environmental gains; (v) tabulate information identifying the research; (vi) perform economic and environmental analysis before and after PPC interference in the production process; (vii) complete the study.

### 3.3. Data Analysis

To analyze the environmental impacts resulting from the resources used in a given manufacturing process or product, the methodology developed by the Wuppertal Institute, which uses the MIPS (Material Input per Service Unit) concept, will be used. This approach can assess the environmental changes associated with the extraction of natural resources and ecosystems. Therefore, it specifies the total amount of resources used for a product throughout its life cycle [49]. MIPS determines the consumption of resources from the moment of nature extraction until the final disposal of the product at the end of its useful life. MIPS calculations were performed as suggested by many authors [4,49,50,51].

This calculation is based on the Material Intensity Factor (MIF) per compartment, with the following divisions: biotic (set of all living organisms), abiotic (set of elements that do not involve living beings), water, and air [52]. As a result, MIPS is obtained by multiplying the mass of the resource by the respective MIF per compartment, according to Equation (1).

(1) $$MIPS=Mass\times {MIF}_{Biotic}+Mass\times {MIF}_{Abiotic}+Mass\times {MIF}_{Water}+Mass\times {MIF}_{Air}$$

The environmental impact of the selected manufacturing process before and after the implemented PPC actions was calculated using the Mass Intensity Factor (MIF) values per unit of resource employed, as shown in Table 2 [49].

To determine the environmental impact, the actual consumption of resources and the respective factors of the MIF shown in Table 2 were considered in Equation (1) before and after the implementation of the actions performed by the PPC.

In addition, it was calculated the carbon emission reductions in the process, according to Equation (2).

(2) $$t{CO}_{2eq}=MWh\times 0.04998\left(according\;to\;the\;Brazilian\;energy\;matrix\right)$$

As an analysis in the evaluation of the economic impact, the savings resulting from the interventions of the PPC in the production process were calculated. Such savings were assessed in relation to the investments made, and their viability was assessed by calculating the return on investment in terms of internal rate of return (IRR) and the discounted payback period. The calculation is performed over periods, and the total sum of the periods needs to be equal to the initial investment value for the debt to be paid off. The calculation of ROI is done by Equation (3) below, and it is worth noting that the lower the value found, the better for the financial health of the company.

(3) $$ROI=\frac{Profit\;after\;taxes}{investment}\times 100$$

The interpretation of the NPV value calculation result, as per Equation (4) below, allows concluding that the investment made will have a better return than the opportunity costs of funds, with positive results (>0). It is important to mention that this formula considers the net cash flow during a single period (Rt), as well as considers the interest rate or discount rate (i). Finally, the IRR calculation can be done by this equation considering the interest rate equal to zero.

(4) $$NPV=\sum \frac{Rt}{{\left(1+i\right)}^{t}}$$

## 4. Case Study

In this item, the results obtained with the analysis of the case studied in this work will be presented. Firstly, the information obtained during the field visits with their respective economic and environmental assessments will be reported.

The company object of the study in this work is a medium-sized company, located in the Metropolitan Region of São Paulo and with average annual gross operating revenue of US$ 18 million. It has 147 employees and operates in the polymeric packaging sector.

**Figure 1:**
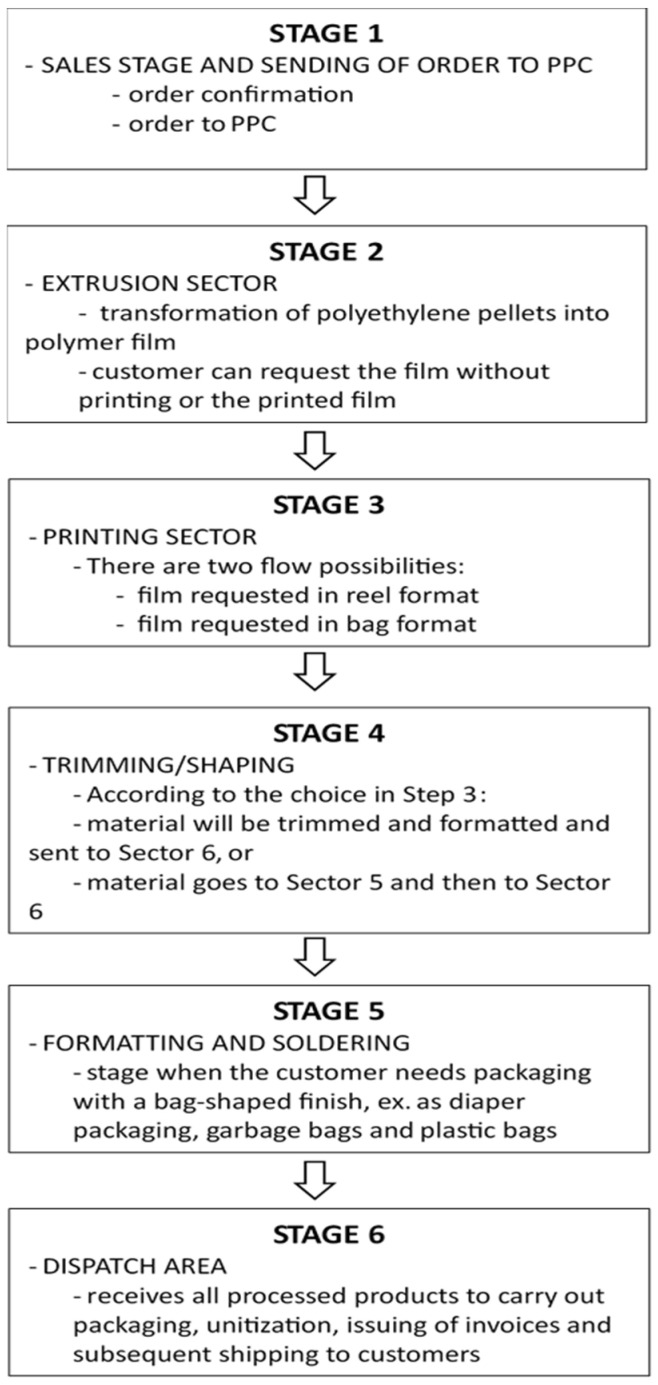
Mapping the production process of company “A”.

Mapping the production process of company “A” involves the following steps, according to Figure 1:

Stage 1. The manufacturing process starts after confirmation of the order negotiated between the customer and the seller. The sales professional issues a pre-order for the PPC sector. This, in turn, launches it in the MRP and generates the need for raw material in order to carry out the planning, preparing the purchase of materials if necessary. After this procedure, a shop order is issued and sent to the first productive sector.

Stage 2. Extrusion sector, which has four extruders with the capacity to process 15,000 kg/day, operating 24 h a day. In this sector, the transformation of polyethylene pallets into a polymeric film takes place. Here, the customer can request film without printing or printed film: (a) If requested, the film without printing, also called “blank”, will have no more steps in the production process, so it is already finalized in the extrusion; (b) if the customer has chosen to obtain the printed film, a manufacturing order is sent to the printing area;

It is important to note that each equipment was considered to be operating under “full operational conditions”; therefore, the manufacturing parameters were deemed sufficient both before and after the modifications in the PPC system. As such, the analysis focused on production-related parameters, such as cycle time and production flow.

An effort was made to distinguish manufacturing variables from production variables—the latter being the central focus of this study. Accordingly, dynamic variables such as temperature, pressure, feed rate, and cooling, as well as static variables like product thickness and die shape (which are related to product specifications), were maintained constant in order to isolate the effects of production variables. This approach was made feasible by ensuring continuous monitoring and control of dynamic variables, keeping their variability to a minimum.

For instance, the melting temperature of the pellets was maintained within the range of 200–220 °C; pressure was kept between 15 and 22 MPa for LLDPE and between 11 and 14 MPa for LDPE. The feed rate was held constant, with the same value applied before and after the PPC intervention, resulting in an average processing capacity of 9 tons per day. The increase in production—from 4.077 to 4.579 tons per year—was primarily achieved, in terms of process parameters, through the reduction of scrap enabled by the modifications in the PPC system.

Stage 3. It is related to the Printing sector, which has two flexographic printer machines with average speeds of 300 m/min and 140 m/min, providing an average of 9 tons/day. At this stage of the production process, there are two flow possibilities: (a) requested film in coil format; (b) requested film in a bag format.

Stage 4. Trimming/shaping, where three machines produce an average of 7 tons/day each. In case of option “a” in step 3, the material will be trimmed and shaped according to the width requested by the customer and later sent to the dispatch area. If the customer’s option is “b”, the material goes to the shaping and welding sector for the closing of the bags, counting, and packaging, which is also carried out in this sector. Afterwards, it is sent for dispatch.

Stage 5. This sector has six machines for shaping and welding, producing 6.5 tons/day. The sector is activated when the customer needs packaging with a bag-shaped finish, such as diaper packaging, garbage bag, plastic bags, among others.

Stage 6. The dispatch area receives all the products processed to pack, unitize, invoice, and then forward to customers.

In the recent past, the company performed stages 1 and 2 of the production process operating in a make-to-stock (MTS) environment. In these conditions, it was common for coils to remain idle for a long time, awaiting orders that matched its width and thickness dimensions. Often, an order was received for the same thickness, but with a smaller width. In such situations, a wider coil was used, generating waste of material. In view of this, the PPC promoted a change in this part of the production system, starting to operate in a make-to-order (MTO) environment, that is, producing the coils only after receiving a firm order. This change made it possible to reduce the stock of extruded coils, eliminating waste and higher costs with materials stopped in stock.

The transition between MTS and MTO systems required the design of a new production system. Initially, this was carried out using the existing equipment (which was later replaced with upgraded machinery after further analysis). It is important to note that, at first, the manufacturing system remained unchanged (utilizing the equipment already installed in the plant).

Thus, the reorganization of the production logic (in light of PPC) enabled the gradual phase-out of the MTS system, while simultaneously implementing the MTO approach. As an immediate result, there was a reduction in the inventory present within the process, leading to a lower amount of work-in-process material. This, in turn, resulted in less capital being tied up (lower production costs) to maintain operations. With optimized production and the implementation of the MTO philosophy, it was also possible to reduce the product’s total production time (cycle time).

The industrial manager mentioned in an interview that production in the shaping/welding sector was far below expectations, producing only 4.5 tons per day due to the excessive stoppage of equipment for maintenance. This situation led the PPC to implement preventive maintenance in all sectors, but in particular with greater rigor in the cutting and welding sector due to the older machines. The action resulted in an improvement in productivity and also a reduction in unusable materials (scrap).

The printing sector’s planning before the PPC’s interference planned the sequencing of production orders (shop orders) by delivery date, without taking into account the characteristics of the production batches. Such a procedure required constant interruptions for the complete setup of the equipment, involving an excessive number of idle hours. The PPC recommended the process change with the materials grouping, taking advantage of characteristics of similar materials, such as printing the internal printing films first and then those of external printing, as this constant inversion in the sequencing demands greater setup time. With this new approach and training of operators, the setup was reduced on average from 5 h to 1.5 h/order, generating electricity savings.

Another sector that formerly based its planning on delivery dates and started to work by grouping and similar characteristics of materials was the extrusion sector. For example, LLDPE (Linear Low-Density Polyethylene) must receive a temperature higher than LDPE (Low-Density Polyethylene) film to be extruded, that is, the constant exchange between these materials generates waste of time and material. The PPC also adopted a standard for the extrusion of films according to their width and thickness, working on the sequencing of the largest widths for the smallest and the thickest thickness for the thinnest. In this way, the PPC standardized a methodology for programming printers and extruders in order to reduce setup time, reducing waste in time and scrap and consequently reducing electricity consumption.

Hyvärinen [5] and Small [6] discuss the use of plastic polymers in the extrusion process. The extrusion of linear low-density polyethylene (LLDPE) and low-density polyethylene (LDPE) is a technique used for manufacturing plastic films, tubes, and other polyethylene products. The process involves transforming the polymer into the specified shape using thermal processing (heat) and pressure. Both LLDPE and LDPE are provided in the form of pellets or granules. The pellets are fed through a hopper into the feed throat of the extruder. The process begins when the material is transported into the extruder barrel. The extruder contains a rotating screw that moves the pellets into a heated barrel. As the material passes through the barrel, the temperature is increased, primarily by the heat supplied in the hot zone of the machine.

The heating/working temperature ranges from 180 °C to 240 °C, and the pellets melt, becoming fluid, which is a key characteristic for the employed process. The molten material is forced through a die to take on the desired shape, resulting in the final product—in this case, plastic films. At this point, the product is still at a high temperature, so rapid cooling is necessary to preserve the final shape. Once the material is cooled, it can be either wound or cut, depending on the need. The plastic films are wound into rolls, which are then sent to industries that will use them to manufacture final products. Polyethylene performs well during the extrusion process due to its flow properties, making it possible to produce extruded products with high specification levels.

The most significant variables in the process are speed (screw and extrusion), temperature (equipment and material), pressure, feed rate, cooling, product thickness, and die shape. Thus, the dynamic variables (temperature, pressure, speed, feed rate, and cooling) must be monitored and controlled in real time, as variations outside these parameters can result in defects in the final product.

Regarding the differences between LLDPE and LDPE, the key factors for processing are their structure and melting point. LDPE is less dense, which makes it more flexible compared to LLDPE. On the other hand, the structure of LLDPE is more “organized”, which results in a material with higher resistance properties, especially in terms of tensile strength. Considering the melting point, LDPE has a lower melting point compared to LLDPE, which, at first glance, may result in lower energy consumption for processing.

As shown by the descriptions above, the implementation of these actions led the company to make economic gains in its production system. However, the company had a bottleneck that hindered the fulfilment of orders within the correct deadlines, due to operational problems in the extruders. A study developed jointly by Manufacturing Engineering, Maintenance and PPC areas concluded that it would be feasible to exchange two old extruders for two new ones, providing an increase in productivity in the process and a reduction in electricity consumption. That way, the new extruders went from an average of 11 tons/day to 15 tons/day, increasing their productivity by 36.4%. The investment in these two new extruders totaled approximately US$ 700 thousand. The old machines could still be sold for around US$ 150 thousand, which meant that the company’s net disbursement was US$ 550 thousand.

Table 3 summarizes the economic gains resulting from the actions implemented in PPC. It can be observed that the contribution margin increased from 6126.9 to 6881.3, that is, by 754.4. This result was achieved due to the reduction in the variable cost of the process, as the lower waste rate and shorter cycle time led to a decrease in the production cost per unit.

The information obtained from the studied company made it possible to calculate the savings generated as a result of implementing the suggestions for changes in the production process arising from the PPC. This calculation is detailed in Table 3.

When analyzing the economic results before and after the PPC’s interference in the production process, it was possible to notice an increase in the company’s contribution margin of US$ 754 thousand per year. On the other hand, as there was a reduction in the volume of polymer scrap, the company lost US$ 8.5 thousand as a result of the decrease in revenue received due to the sale of that material. Finally, the electricity savings generated an annual savings of US$ 73 thousand, which allowed a total economic advantage resulting from the PPC’s actions of US$ 819 thousand per year.

As noted above, in order for these values to be achieved, the studied company invested around US$ 550 thousand. The feasibility analysis of this investment showed that the annual savings of US$ 819 thousand generated an internal rate of return (IRR) of 96% per year and a discounted payback period (15% per year discount rate) of 14 months. This result clearly shows the contributions that the PPC can make to improve the economic performance of a company. In fact, if instead of dedicating itself exclusively to its traditional functions of planning, scheduling, and controlling production, the PPC also has a more strategic view of the business insofar as it is predisposed to also act in the search for cost reductions and increases revenue, the PPC may become a more relevant function than it has traditionally been.

However, it is not only in improving economic results that the PPC can contribute. Its performance also brings reductions in the environmental impact generated by industrial operations, as shown by Oliveira Neto [4].

To carry out the environmental analysis calculation, the kilogram was considered as the unit of measurement and LLDPE and electric energy as production inputs. For this purpose, annual reductions in the consumption of the considered inputs were obtained from the company, assuming a standard production quantity. Then, the environmental impact calculation was performed using the volume of resources multiplied by the respective values of their Mass Intensity Factors (MIF), according to Wuppertal [53]. Table 4 shows the details of the calculations made.

In addition, it was calculated the carbon emission reductions in the process, according to Table 4. The reduction in electricity consumption resulting from the implementation of actions in PPC amounted to 611,364 kWh per year, decreasing from 4,367,638 kWh per year to 3,756,274 kWh per year. Thus, considering the conversion factor for the Brazilian energy matrix of 0.04998 metric tons of carbon equivalent (tCO_2_eq) per megawatt-hour, this corresponds to approximately 30.57 metric tons of avoided carbon emissions.

As shown in Table 4, the actions implemented by suggestions from the PPC allowed a reduction of 30,631 tons per year in the use of resources taken from nature and carbon emission reductions in the process of 30.57 tons per year. This point clarifies that the more proactive role of the PPC, transcending its traditional functions, not only allowed for significant economic gains but also generated an important reduction in the environmental impacts resulting from the company’s operations. In other words, this work by the PPC contributes to increasing the level of eco-efficiency of the studied company.

## 5. Discussion

The studied company carried out the recovery and reuse of chips, demonstrating that it is an activity that can provide both economic and environmental gains.

This result demonstrated alignment with the literature regarding the adoption of recovery of products or raw materials by the PPC, as shown by Mawandiya [38] through the recovery and reuse of automotive batteries (acid-lead) in a closed cycle to minimize the environmental impact and promote economic gain. Prajogo [35] also emphasized the need for the PPC to reduce waste through recovery and reuse of raw material. Lage [36] presented a mathematical model in order to minimize the total cost of recovering materials in the remanufacturing process. Digalwar [33] considered the PPC as a performance measure aimed at recovering materials and introducing cleaner technologies in production, while Munot [34] mentioned that remanufacturing aims to restore products used in remanufactured products with such good-quality conditions as for new products.

Thus, for Jabbour [13], the adoption of the eco-efficient PPC can occur as waste is reduced through recycling, reuse, or remanufacturing. What can be concluded is that these activities identified in the field research developed here contributed to the literature and to the practice by demonstrating the possibility of companies in the polymer sector to use recovery and reuse for not only economic but also environmental benefits. It is also possible to mention that scrap recovery and reuse was the PPC activity that had the strongest relationship with eco-efficiency.

Another finding was that load sequencing favours eco-efficiency, as it reduced waste of time and scrap. However, load sequencing is an activity that, if not well-executed, negatively impacts eco-efficiency. In the studied company, for example, the sequencing activity had a very strong repercussion, due to the fact that the PPC professional works only with one sequencing criterion. As noted, this is an activity that requires flexibility and knowledge and cannot be cast on just one criterion as was previously practiced (only the delivery date as a cargo sequencing criterion). This attitude generated a larger amount of chips since the work was made with abrupt changes constantly, instead of working with groups of similar materials. The company only started to obtain economic and environmental gains when it changed the way of sequencing and started to group similar service orders and use materials, such as printing on the outside or inside of the polyethylene film. The alternation between internal and external printing is an abrupt change that requires more setup time and a consequently greater waste of chips. In this way, the longer the alternation time of these two types of impressions is possible, the better for reducing waste. With that, the company started to carry out cargo sequencing by grouping shop orders. For example, it was only when the batches with external printing were finished that the setup of the internal prints was initiated by means of another grouping, in order to take advantage of the conversion and run all the internal printing OFs that they had on closer delivery dates.

This result corroborates the literature insofar as Cannata [16] mentioned the importance of the activity to reduce the consumption of electricity through sequencing. It also contributes to the research by Plehn [18], who measured the reduction in setup and electricity by 13%, and with the work of Oliveira Neto and Lucato [4], who reduced production costs by 42% and reduced environmental impact by 13%, using load sequencing. Therefore, it is an activity that, as demonstrated both in the literature and in in-field research, can provide improvement in eco-efficiency.

The reduction in the setup is another activity that provided eco-efficiency improvements by reducing the setup time on average by 70% on the company’s printers. This result is in line with the teachings of Shingo [54] through the application of four phases: strategic, preparatory, operational, and proof. It is worth noting that the 70% setup reduction occurred because some PPC strategies were implemented. Initially, process planning and programming were organized to minimize setup changes, grouping similar products to reduce the frequency of changes. Afterwards, the processes were standardized, in terms of tool changes and adjustments, ensuring that all operators follow the same steps. At the same time, it was necessary to implement on-the-job training so that all operators know how to perform changes quickly and efficiently, reducing downtime. Thus, PPC added setup time analysis to the process performance indicators to control the time spent in each setup stage, which allowed waste to be identified and eliminated.

Another relevant finding was that preventive maintenance provided more eco-efficiency in the studied company, denoting that in addition to preventing unwanted stops due to wear of parts, it avoided the generation of chips, since each time the machine was stopped, 10 to 20 kg of chips was generated on average. The company demonstrated the importance of carrying out this activity since until then it had only corrective maintenance. After the analysis described above, the opportunity to obtain economic gains with the implementation of preventive maintenance practices was found and these were planned by the PPC, which has production control throughout the manufacturing floor. Normally, this activity should be performed during periods of machine idle so as not to hinder production planning in terms of waste of time and trim. Thus, the use of preventive techniques by the PPC with a focus on environmental proactivity is an embryonic aspect that generates eco-efficiency gains. Preventive actions are related to environmental proactivity, making it possible to reduce waste in the use of raw materials, electricity, water, and various by-products. This finding innovates the PPC literature, suggesting further scientific research on the subject.

No work has been found in the literature that mentions economic and environmental gain by switching from MTS to MTO systems. However, in the case study presented, the system change was performed, since the plastic coils that were made for stock were not always used correctly. In this way, when there were no shop orders with the same dimensions, the coils ended up becoming obsolete due to the long storage time. The result was the need to cut them, making more excessive scrap.

It is noteworthy that generally the works that link the PPC with eco-efficiency present only qualitative evidence about the possible environmental gain. Only Oliveira Neto and Lucato [4] carried out an environmental impact assessment in the area of PPC in the chemical sector. However, the research developed here also contributed to the literature through the presentation of quantitative data that represented a reduction in the environmental impact on ecosystems, however in companies that manufacture polymeric packaging.

The studied companies also obtained economic gain through the activities of PPC. Researches that linked PPC to eco-efficiency in general showed qualitative evidence that can provide economic gains. The only survey that found quantitative data to analyse economic gains was the research by Oliveira Neto and Lucato [4] carried out in the chemical sector. However, what differs the present research from the research just mentioned is the fact that it was carried out in polymeric packaging manufacturers, thus contributing to the literature as it was developed in another productive sector.

From a technical standpoint, the principles of PPC are applicable to manufacturing facilities of any size or scale. However, local specificities and operational requirements play a critical role in determining the necessary adjustments to enhance operational efficiency—one of the core objectives of PPC. While production scale may be a relevant factor in contexts similar to the one examined in this study, greater challenges to process optimization typically arise from the nature of the production system and the diversity of products manufactured. It is essential that manufacturing variables remain within specified control limits, as these parameters are directly associated with the overall performance and efficiency of the production system.

## 6. Conclusions

This work presented a case study connecting the activities of the PPC to eco-efficiency. The study indicated that the PPC’s proactive action, in addition to its traditional functions of planning, programming, and controlling production, can contribute to the company in the identification and implementation of economic and environmental gains.

Thus, the objectives of this work were achieved by demonstrating the confirmation of the suggested proposition that the PPC, through its activities/tools integrated with environmental practices, can promote improvements in eco-efficiency in companies that manufacture polymeric packaging. These activities are recovery and reuse, load sequencing, reduction of setup, preventive maintenance, and system switch from MTS to MTO.

The adoption of Production Planning and Control (PPC) strategies in the packaging industry has proven to be crucial in reducing polymer waste. Optimizing the extrusion process through PPC enables more efficient use of materials by adjusting critical variables in real time, such as temperature and pressure, which significantly reduces waste. Additionally, PPC practices not only generate economic benefits but also contribute to sustainability by reducing the environmental impact associated with polymer production. With the increasing demand for more eco-friendly solutions, the implementation of these strategies leads to significant progress in the circular economy model, promoting recycling and resource reuse, which is essential for fostering a more efficient and environmentally responsible industry.

This study brought contributions to theory, practice, and society. From a theoretical point of view, the results obtained here innovate in that they add to the existing literature a study that demonstrates the possibility of the PPC acting in the generation of economic and environmental advantages in the polymeric packaging industry. A similar study was carried out by Oliveira Neto [4] but in the chemical industry, in a different treatment from the one studied in this article. For PPC managers and practitioners in the polymeric packaging industry, this study provides a clear example of how a more creative and proactive approach to PPC activities can bring economic and environmental gains to the company. Finally, the presented case study brings contributions to society as the environmental gains generated a decrease of 30 thousand tons of resources extracted from nature, which contributes to guarantee the activities of the present, minimizing the impact for future generations.

However, as it is common in researches, it also has some limitations. Initially, this study does not allow generalization of results due to the use of a single case. That leaves the door open for an expansion of the present study, either by developing more exploratory case studies as a knowledge base to carry out more comprehensive future research with generalization power. For example: through a survey.

Another limitation may be associated with the possibility of replacing the MTS system for the MTO. In fact, in principle, the MTO is a system that all companies would like to adopt. However, there are other market demands, such as service levels. Thus, there is much to be explored to analyze the pros and cons and how interesting it would be for a company to make this system change. As demonstrated in this work’s case study, the exchange of MTS for MTO could improve the company’s eco-efficiency level, generating economic gains and a reduction in the environmental impacts in polymeric packaging companies. The data used for the calculations are not estimates; they are real based on the process, and the pertinent calculations are explained in the research methodology. Therefore, the data are not statistical; they are real numbers collected at the plant of the company researched.

## Figures and Tables

**Table 1:** Selected papers relating PPC and eco-efficiency.

Group	Reference	Journal
1. Recycling, Reuse, and Remanufacturing	[25]	*Production Planning & Control*
	[26]	*Robotics and Computer Integrated Manufacturing*
	[27]	*Journal of Operations Management*
	[28]	*Institute for Operations Research and the Management Sciences*
	[29]	*Production Planning & Control*
	[30]	*International Journal of Production Research*
	[31]	*International Journal of Production Economics*
	[32]	*International Journal of Sustainable Engineering*
	[19]	*Production Planning & Control*
	[33]	*Measuring Business Excellence*
	[34]	*Journal of Mechanical Engineering and Sciences*
	[35]	*International Journal of Operations & Production Management*
	[36]	*Central European Journal of Operations Research*
	[37]	*International Journal of Sustainable Engineering*
	[38]	*International Journal of Sustainable Engineering*
2. Water and Energy Efficient Usage	[39]	*Computers & Chemical Engineering*
	[14]	*Production Planning & Control*
	[21]	*Civil Engineering and Environmental Systems*
	[15]	*International Journal of Operations & Production Management*
	[40]	*Computers and Chemical Engineering*
	[18]	*CIRP Annals—Manufacturing Technology*
	[41]	*International Conference on Production Research*
	[42]	*International Journal of Computer Integrated Manufacturing*
	[43]	*International Journal of Sustainable Engineering*

**Table 2:** Material Intensity Factors (MIF) used in this work.

Resource	Abiotic Materials (Kg)	Biotic Materials (Kg)	Water (Kg)	Air (Kg)
Electric Energy (Kwh)	2.67	0.00	37.9	0.640
LLDPE (Linear Low-Density Polyetthylene)	2.12	0.00	2.80	162.1

Source: Wuppertal [53].

**Table 3:** Economic gains resulting from PPC action implementations.

	PPC Actions		Results
Values in US$ 1000/Year	Before	After	
Production output (ton/year) *	4077	4579	
Net sales billed	11,782.5	13,233.3	
Contribution margin	6126.9	6881.3	754.4
Scrapt sales	37.5	29.0	−8.5
Electric energy consumption	779.5	706.2	73.3
	Total		819.2

* Production capacity limited by extruders that operate 24 h/day.

**Table 4:** Calculation of the environmental impact reduction resulting from the actions of the PPC.

	PPC Actions			Compartments				
	Before	After *	Reduction	Abiotic	Biotic	Air	Water	
LLDPE consumption (kg/year)	149,059	116,545	32,514	2.12 68,930	0 0	2.8 91,039	162.3 5,277,022	MIF (kg/kg) Environmental impact (kg/year)
Electric energy consumption (kWh/year)	4,367,638	3,756,274	611,364	2.67 1,632,342	0 0	37.9 23,170,696	0.64 391,273	MIF (kg/kWh) Environmental impact (kg/year)
Environmental impact reduction per compartment (kg/year)				1,701,272	0	23,261,735	5,668,295	
Total environmental impact reduction (t/year)				30,631				
Carbon dioxide equivalent emissions (tCO_2eq_) **	218.22	187.65	30.57					

* Consumptions after PPC actions adjusted for the same “before” production volumes. ** tCO_2eq_ = MWh × 0.04998 (according to the Brazilian energy matrix).

## Data Availability

Data set available on request from the authors.
